# O-GlcNAcylation levels remain stable regardless of the anaesthesia in healthy rats

**DOI:** 10.1038/s41598-024-61445-0

**Published:** 2024-05-09

**Authors:** Thomas Dupas, Amandine Vergnaud, Thomas Pelé, Angélique Blangy-Letheule, Virginie Aillerie, Martin Bouaud, Angélique Erraud, Anaïs Maillard, Dorian Hassoun, Antoine Persello, Jules Lecomte, Matthieu Rivière, Arnaud Tessier, Aurélia A. Leroux, Bertrand Rozec, Manon Denis, Benjamin Lauzier

**Affiliations:** 1 INSERM, L’institut du thorax, CNRS, Nantes Université, 8 Quai Moncousu, 44007 Nantes, France; 2 INSERM, L’institut du thorax, CHU Nantes, CNRS, Nantes Université, 44000 Nantes, France; 3 Faculté des Sciences et des Techniques, Chimie et Interdisciplinarité: Synthèse, Analyse, Modélisation (CEISAM), UMR CNRS 6230, CNRS, Université de Nantes, Nantes, France; 4 Oniris, 44300 Nantes, France

**Keywords:** Anaesthesia, O-GlcNAcylation, Post-translational modification, Metabolism, Therapeutic strategy, Post-translational modifications, Homeostasis

## Abstract

Anaesthetics are used daily in human and veterinary medicine as well as in scientific research. Anaesthetics have an impact on cell homeostasis especially through modulation of protein post-translational modifications. O-GlcNAcylation, a ubiquitous post-translational modification, plays a role in many biological processes. The aims of this study were to evaluate whether (1) anaesthesia influences O-GlcNAcylation and (2) its stimulation affects physiological parameters. Male Wistar rats (n = 38) were anaesthetized with ketamine-xylazine or isoflurane. They randomly received either an intravenous injection of Ringer's lactate or NButGT (10mg/kg) in order to increase O-GlcNAcylation levels. One hour after induction of anaesthesia, haemodynamic parameters and plasmatic markers were evaluated. Heart, brain and lungs were harvested and O-GlcNAcylation levels and O-GlcNAc-related enzymes were evaluated by western blot. Cardiac and pulmonary O-GlcNAcylation levels and cardiac, cerebral and pulmonary O-GlcNAc associated enzyme expression were not impacted with anaesthesia. Compared with ketamine-xylazine, isoflurane had a lower impact on blood pressure, heart rate and glycaemia. Pharmacological stimulation of O-GlcNAcylation by NButGT did not affect the physiological parameters. This study offers unprecedented insights into the regulation of O-GlcNAcylation and O-GlcNAc related enzymes during anaesthesia. Pharmacological stimulation of O-GlcNAcylation over a 1-h period did not disrupt the physiological balance in healthy anaesthetized rats.

## Introduction

Anaesthesia is achieved by reversible inhibition of the central and peripheral nervous system, effectively mitigating the neurovegetative responses associated with pain and nociceptive stimulation^1,2^. General anaesthesia induces a loss of consciousness and is used worldwide for surgery (e.g. knee replacement, open-heart surgery) as well as in research on laboratory animals. Intravenous and inhaled anaesthetic agents are both commonly used in medicine and in research. Among the numerous anaesthetic agents available, ketamine and isoflurane stand out as the most commonly used^3,4^. Ketamine, a noncompetitive antagonist of *N*-methyl *D*-aspartate (NMDA) receptors, induces amnesia, analgesia and immobility. It is frequently used in combination with xylazine, an α2 adrenergic agonist, that not only enhances analgesia but also provides muscle relaxation and sedation^5^. Isoflurane is a volatile anaesthetic agent which acts by inhibiting NMDA, gamma-aminobutyric acid and glycine receptors, resulting in amnesia, sedation and immobility^6^. Despite these distinct modes of action, anaesthesia has been recognized for its influence on protein post-translational modification (e.g. phosphorylation, ubiquitinylation, methylation), associated with changes in the biological process including heart contractility, cytoskeleton remodelling and synaptic plasticity and memory^7–13^. However, its full spectrum of action remains incompletely understood.

O-GlcNAcylation (O-GlcNAc) is a post-translational modification which occurs on serine and threonine from more than 5000 proteins in humans^14^. Uridine diphosphate N-acetylglucosamine (UDP-GlcNAc) is produced through the hexosamine biosynthesis pathway (HBP) and used as a sugar donor by the O-GlcNAc transferase (OGT) to O-GlcNAcylate proteins, while O-GlcNAcase (OGA) removes GlcNAc moiety. Glutamine-fructose-6-phosphate aminotransferase (GFAT) is the rate-limiting enzyme of the HBP and exists in two isoforms, GFAT1 and GFAT2^15^. O-GlcNAcylation is ubiquitous and can modify nuclear, cytosolic, mitochondrial and extracellular proteins, thereby explaining its role in a widespread range of signalling pathways and therefore in many, if not all, biological processes^14,16^.

Previous studies have suggested a relationship between this protein modification and anaesthesia. In fact, the use of isoflurane significantly increased UDP-GlcNAc levels in the prefrontal cortex and increased myocardial proteins O-GlcNAcylation levels in mice^17–19^. However, assessment of the impact of anaesthesia on O-GlcNAcylation was not the primary objective of these studies, contributing to the ambiguity of the obtained results. Evaluation of the effect of anaesthetic agents on this protein modification is even more important considering that acute stimulation of O-GlcNAcylation could be beneficial in clinical situations requiring general anaesthesia (e.g. cardiac surgery) The aims of this study were (1) to determine if anaesthesia affects O-GlcNAcylation levels and O-GlcNAc-related enzymes and (2) to determine if pharmacological stimulation of O-GlcNAcylation could affect the physiological parameters in anaesthetized healthy rats.

## Results

### Impact of anaesthesia on O-GlcNAcylation and hexosamine biosynthesis pathway

**Figure 1:**
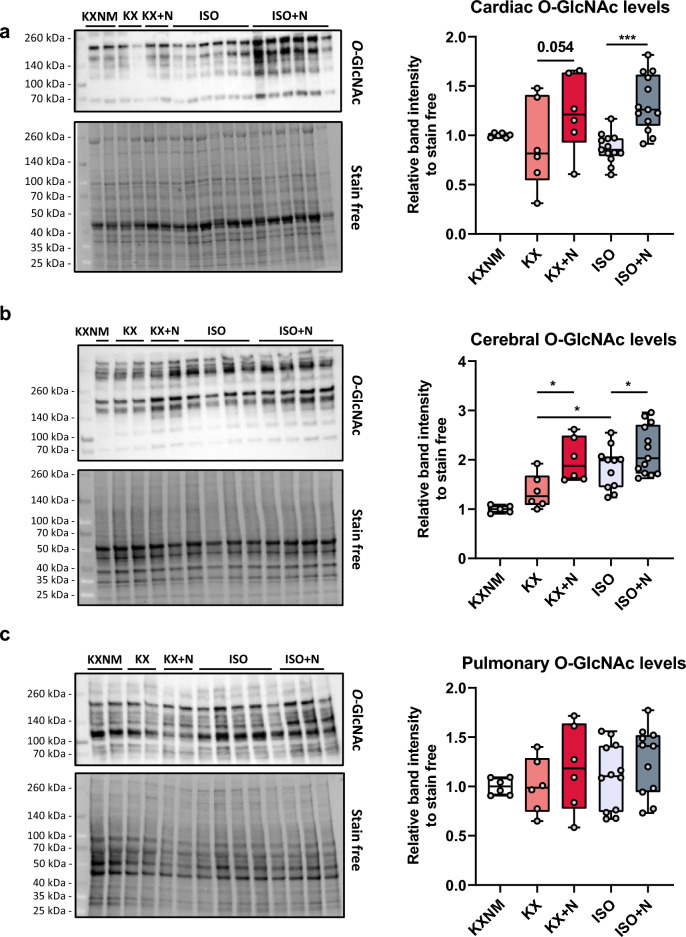
NButGT increases O-GlcNAcylation levels in a tissue-specific manner. Cardiac (**a**), cerebral (**b**) and pulmonary (**c**) global protein O-GlcNAcylation levels were evaluated on rats under non-maintained anaesthesia (KXNM), ketamine–xylazine-maintained anaesthesia (KX), isoflurane anaesthesia (ISO), supplemented with NButGT (KX + N; ISO + N) to stimulate O-GlcNAcylation levels. Quantification of western blots are related to stain free. The top of the box represents the 75th percentile, the bottom of the box represents the 25th percentile, and the line in the middle represents the 50th percentile. The whiskers represent the highest and lowest values. *p < 0.05; ***p < 0.001; KXNM (n = 5–6); KX (n = 6); KX + N (n = 6); ISO (n = 11–13); ISO + N (11–13). Full blots are shown in Supplementary Fig. S1.

We first assessed the effect of 1-h anaesthesia on cardiac, cerebral and pulmonary O-GlcNAcylation levels and O-GlcNAc-related enzymes. The maintenance of anaesthesia did not impact the levels of O-GlcNAcylation in the three organs studied (Fig. 1; Supplementary Fig. S1). Cardiac O-GlcNAc levels were not impacted by anaesthesia type. NButGT increased O-GlcNAcylation levels in the heart (KX: 0.90 ± 0.18; KX + N: 1.22 ± 0.16; ISO: 0.87 ± 0.04; ISO + N: 1.32 ± 0.08; p < 0.05) (Fig. 1a). Cerebral O-GlcNAc levels increased with isoflurane anaesthesia compared with ketamine-xylazine (KX: 1.36 ± 0.14; ISO: 1.83 ± 0.14; *p* < 0.05). NButGT injection increased O-GlcNAc levels in the brain, independently of the anaesthesia used (KX: 1.36 ± 0.14; ISO: 1.83 ± 0.14; KX + N: 2.00 ± 0.18; ISO + N: 2.19 ± 0.14; p < 0.05) (Fig. 1b). Pulmonary O-GlcNAc levels were unmodified in all of the studied groups, even with NButGT treatment (Fig. 1c). Cardiac OGT increased in the ISO + N group compared with KX + N, while its expression was unchanged in the brain and the lung (KX + N: 0.45 ± 0.05; ISO + N: 0.70 ± 0.22; *p* < 0.05). In the brain, OGA decreased in the ISO + N group compared with the KX + N and ISO groups (KX + N: 1.54 ± 0.22; ISO + N: 1.11 ± 0.09; *p* < 0.05). Conversely, its expression increased in the ISO + N group in relation to the KX + N group in the lung (KX + N: 1.60 ± 0.72; ISO + N: 2.03 ± 0.53; *p* < 0.05). OGA expression was unmodified in the heart. Expression of GFAT1 and GFAT2 in the heart, brain, and lung remained unchanged in all of the groups (Supplementary Table S1; Supplementary Fig. S2, S3 and S4). All of these data highlight that 1 h after injection, NButGT increased O-GlcNAcylation levels without affecting the expression of O-GlcNAc-related enzymes. The results also suggest that tissue has a different sensitivity to O-GlcNAc modulation and NButGT treatment.

### Haemodynamic parameters were not impacted by O-GlcNAcylation level stimulation

**Figure 2:**
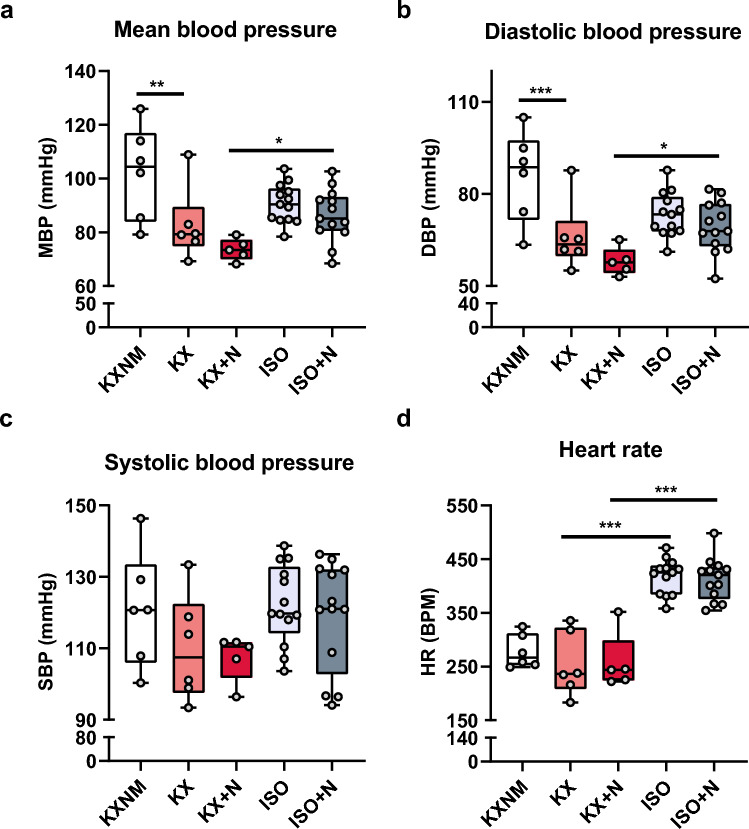
Mean blood pressure and heart rate are impacted depending on the type of anaesthesia. Mean (**a**), diastolic (**b**), systolic (**c**) blood pressure and heart rate (**d**) were evaluated on rats under non-maintained anaesthesia (KXNM), ketamine–xylazine-maintained anaesthesia (KX), isoflurane anaesthesia (ISO), supplemented with NButGT (KX + N; ISO + N) to stimulate O-GlcNAcylation levels. The top of the box represents the 75th percentile, the bottom of the box represents the 25th percentile, and the line in the middle represents the 50th percentile. The whiskers represent the highest and lowest values. *p < 0.05; **p < 0.01; ***p < 0.001; KXNM (n = 6); KX (n = 6); KX + N (n = 5); ISO (n = 13); ISO + N (13).

Haemodynamic parameters undergo changes during anaesthesia. We evaluated the impact of the different types of anaesthesia and pharmacological stimulation of O-GlcNAcylation levels on these parameters. As expected, mean blood pressure decreased with ketamine-xylazine anaesthesia compared with the non-maintained anaesthesia group (KXNM: 102 ± 7; KX: 82 ± 5; mmHg; *p* < 0.05), which can be explained by the drop in diastolic blood pressure (KXNM: 86 ± 6; KX: 66 ± 5; mmHg; *p* < 0.05). Isoflurane-maintained anaesthesia appeared to have less impact on mean, systolic and diastolic arterial pressure compared with ketamine-xylazine-maintained anaesthesia (Fig. 2a–c). Both mean and diastolic blood pressure are affected to a lesser extent in the ISO + N group than in the KX + N group (Fig. 2a, b). Systolic blood pressure is unchanged between the different groups (Fig. 2c). With respect to ketamine-xylazine anaesthesia, isoflurane less impacted the heart rate, regardless of NButGT treatment (KX: 254 ± 24; KX + N: 258 ± 24; ISO: 418 ± 9; ISO + N: 412 ± 11; BPM; p < 0.05) (Fig. 2d). As expected, NButGT injection had no impact on any of the hemodynamic parameters assessed (Fig. 2).

### Blood gases, pH and metabolism were unchanged with O-GlcNAcylation stimulation

**Figure 3:**
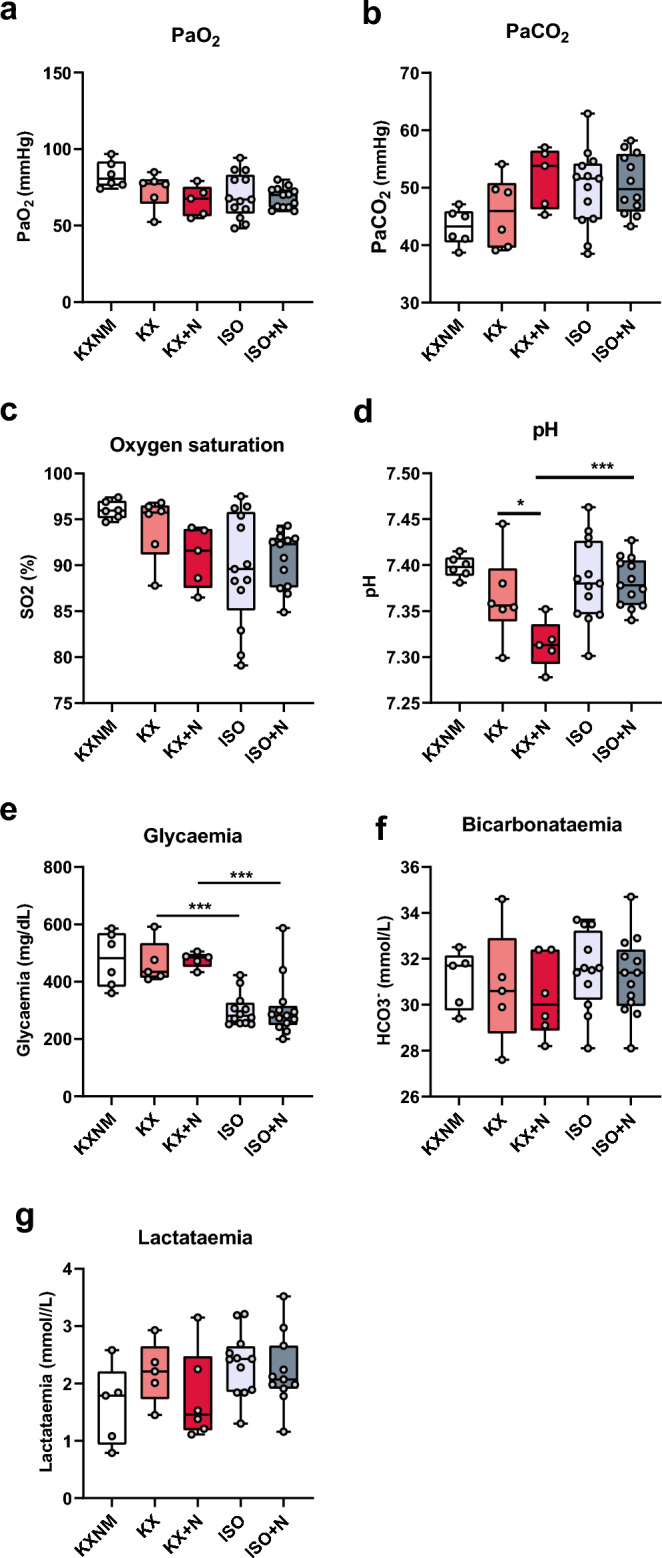
NButGT treatment does not impact blood gazes, pH and metabolism. Arterial partial pressure of oxygen (**a**), carbon dioxide (**b**), oxygen saturation (**c**), venous pH (**d**), bicarbonataemia (**e**), glycaemia (**f**) and lactataemia (**g**) were evaluated on rats under non-maintained anaesthesia (KXNM), ketamine–xylazine-maintained anaesthesia (KX), isoflurane anaesthesia (ISO), supplemented with NButGT (KX + N; ISO + N) to stimulate O-GlcNAcylation levels. The top of the box represents the 75th percentile, the bottom of the box represents the 25th percentile, and the line in the middle represents the 50th percentile. The whiskers represent the highest and lowest values. *p < 0.05; ***p < 0.001; KXNM (n = 6); KX (n = 6); KX + N (n = 5–6); ISO (n = 12–13); ISO + N (11–13).

Ketamine-xylazine and isoflurane are known to induce respiratory depression that could lead to changes in blood gases and pH^20^. Arterial partial pressure of oxygen and carbon dioxide as well as oxygen saturation were unchanged in all of the studied groups (Fig. 3a–c). Venous pH decreased with NButGT injection, only in the ketamine-xylazine group (KX: 7.36 ± 0.02; KX + N: 7.31 ± 0.01; *p* < 0.05) (Fig. 3d). pH changes could not be explained by the venous bicarbonataemia which remained constant in all of the groups (Fig. 3e). Venous glycaemia was less impacted with isoflurane compared with ketamine-xylazine-mediated anaesthesia whether or not treated with NButGT (KX: 466 ± 33; KX + N: 476 ± 12; ISO: 300 ± 16; ISO + N: 307 ± 28; mg/dL; *p* < 0.001) (Fig. 3f). Finally, venous lactataemia remained unchanged in the studied groups (Fig. 3g).

### Ionic balance remained within physiological values in the different groups

**Figure 4:**
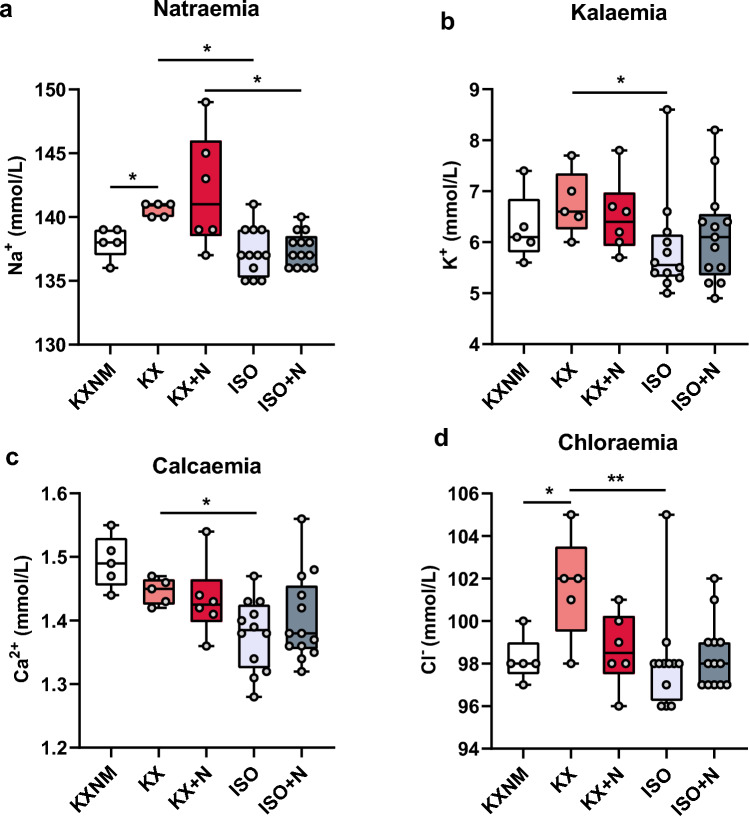
Impact of different types of anaesthesia and stimulation of O-GlcNAcylation on ion balance. Natraemia (**a**) kalaemia (**b**) calcaemia (**c**) and chloraemia (**d**) were evaluated on rats under non-maintained anaesthesia (KXNM), ketamine–xylazine-maintained anaesthesia (KX), isoflurane anaesthesia (ISO), supplemented with NButGT (KX + N; ISO + N) to stimulate O-GlcNAcylation levels. The top of the box represents the 75th percentile, the bottom of the box represents the 25th percentile, and the line in the middle represents the 50th percentile. The whiskers represent the highest and lowest values. *p < 0.05; **p < 0.01; KXNM (n = 5); KX (n = 5); KX + N (n = 6); ISO (n = 12); ISO + N (13).

Ion channels are targeted by anaesthetics and contribute to general anaesthesia^21^. Natraemia increased in the ketamine-xylazine maintained group compared with the unmaintained group. Isoflurane anaesthesia reduced, with or without NButGT, natraemia compared with the KX and KX + N groups (Fig. 4a). Kalaemia and calcaemia were both decreased with isoflurane compared with ketamine-xylazine (Fig. 4b, c). Chloraemia increased in the KX group compared with the KXNM group and decreased in the ISO group compared with the KX group (Fig. 4d). Blood electrolytes were unaffected by NButGT (Fig. 4).

### Impact of anaesthesia and O-GlcNAcylation stimulation on complete blood count

Finally, we assessed the impact of anaesthesia and pharmacological O-GlcNAcylation stimulation on complete blood count. White blood cells decreased in the KX group compared with the KXNM group owing mainly to the decrease in lymphocytes but also in neutrophils and monocytes to a lesser extent. Similarly, white blood cells decreased in the ISO + N group compared with the KX + N group owing to lymphocytes and monocytes. Isoflurane increased eosinophiles compared with ketamine-xylazine, regardless of NButGT. Despite an increase in red blood cells in the KX + N group compared with the KX group, haemoglobinaemia was unchanged in all of the groups. Platelets were also unmodified in the groups (Supplementary Table S2).

## Discussion

In this study, we demonstrated that maintained anaesthesia with either ketamine-xylazine or isoflurane had no impact on cardiac and pulmonary O-GlcNAcylation and related enzymes. We present evidence suggesting that the stimulation of O-GlcNAcylation levels in healthy anaesthetized rats has a moderate effect on physiological parameters, irrespective of the type of anaesthesia.

Despite evidence of a potential link between anaesthesia and O-GlcNAcylation, no studies have been specifically designed to evaluate the impact of the type of anaesthesia on this post-translational modification under standardised conditions. We emphasise that anaesthesia does not affect cardiac or pulmonary protein O-GlcNAcylation levels or O-GlcNAc-related enzymes in the heart, brain, and lungs. Under experimental conditions, (myocardial ischaemia–reperfusion in mice) Hirose et al. reported that isoflurane increased cardiac O-GlcNAcylation levels^19^. This discrepancy may be partly due to the fact that our study was specifically designed to evaluate O-GlcNAc levels under anaesthesia with no other insult. Moreover, the antibody used for O-GlcNAc detection (RL2 in our study; CTD110.6 in Hirose et al*.*) could detect a different or nonspecific target. Indeed, these two most common pan-O-GlcNAcylation antibodies recognise different GlcNAc residues on proteins^22–24^. CTD110.6 has been described as cross-reacting with N-GlcNAc_2_-modified glycoproteins and displaying poor reactivity toward contractile proteins which dominate the cardiac proteome, thus limiting the interpretation of the results^25,26^. Interestingly, Baer et al. demonstrated that isoflurane increased brain cortical UDP-N-acetylglucosamine levels, the substrate used by OGT^17^. However, we have previously demonstrated that UDP-GlcNAc does not appear to be the main regulator element for O-GlcNAcylation, potentially explaining the lack of variation in O-GlcNAc levels observed in the brain under our conditions^15^.

Stimulation of O-GlcNAcylation levels through pharmacological molecules has been around for several years as a potential therapeutic strategy for acute situations. We and others have previously demonstrated that stimulating O-GlcNAc levels restores blood pressure, heart rate and different circulating parameters in models of septic and haemorrhagic shock^27–29^. In this study, we targeted a similar increase in O-GlcNAc levels (1.5–2-fold higher) to evaluate in healthy anaesthetized rats the impact of anaesthesia on these models. Neither blood pressure nor heart rate were modified with the acute NButGT treatment, suggesting the existence of sensors and/or distinct mechanisms. Moreover, ionic balance, blood gases and glycaemia were also unaffected by acute O-GlcNAc stimulation. All of these data illustrate that the increase in O-GlcNAcylation levels obtained through OGA inhibition within a range of 1.5–2 over a short period (1 h) had no significant effect on these healthy animals.

In our study, ionic concentrations, complete blood count and blood gases remained within physiological limits and were moderately impacted by the type of anaesthesia. In comparison with physiological values without anaesthesia, heart rate and mean blood pressure were less impacted with isoflurane-maintained anaesthesia compared with ketamine-xylazine. These results highlight the cardiodepressant effect of ketamine-xylazine and are consistent with studies which reported that isoflurane had lower haemodynamic effects with respect to ketamine-xylazine anaesthesia^31–33^. Blood glucose levels were also less impacted with isoflurane-maintained anaesthesia than by ketamine-xylazine, which is consistent with previous findings demonstrating hyperglycaemia and reduced plasma insulin levels with ketamine-xylazine^34,35^. Our findings compellingly highlight the advantageous choice of isoflurane over ketamine-xylazine as an anaesthetic for experimental animal research since it has less impact on physiological constants.

We pinpointed that depending on the organ, the pharmacological stimulation of O-GlcNAcylation with NButGT was more or less effective, suggesting a tissue sensitivity to the stimulation of O-GlcNAcylation. In this sense, Nöt et al. demonstrated that 24 h after PUGNAc injection (an OGA inhibitor) in haemorrhagic shock rat, O-GlcNAcylation levels were increased in the lung and unchanged in the heart, highlighting that tissue sensitivity could be dependent on the molecule used and/or the situation^36^. Interestingly, we have previously shown that the regulation of O-GlcNAcylation throughout development is tissue specific, which could potentially explain the difference in tissue response to O-GlcNAcylation stimulation^15^. Pharmacological treatment with NButGT does not affect the expression of the O-GlcNAc-related enzymes 1 h after injection. Similar results were obtained by Ferron et al. in later times in lipopolysaccharides (2 h after injection) and cecal ligation puncture (24 h after injection) rat model^28^. Interestingly, despite that regulatory mechanisms have been described as maintaining O-GlcNAcylation homeostasis in vitro (OGA and OGT expression increased and decreased respectively when O-GlcNAc levels were pharmacologically stimulated)*,* no evidence has demonstrated their existence in vivo^37,38^.

In our study, we sought to evaluate the impact of anaesthesia on overall O-GlcNAcylation levels throughout different organs. We demonstrated that the type of anaesthesia does not alter O-GlcNAcylation levels. This could potentially be explained by the implementation of adaptive mechanisms to maintain O-GlcNAcylation levels within a normal range under physiological conditions. These data, although standardised, do not necessarily reflect what could happen under pathological conditions in which O-GlcNAcylation levels could be modified before anaesthesia.

In summary, we demonstrated that cardiac and pulmonary O-GlcNAcylation and associated enzymes are unaffected by the type of anaesthesia and that stimulating O-GlcNAc levels in healthy anesthetized rats has no significant impact on physiological parameters. This underscores that the positive outcomes observed in models of haemorrhagic and septic shock could be attributed to pharmacological O-GlcNAc stimulation rather than anaesthesia^29,36^. These data also suggest that the observed impacts of anaesthesia on O-GlcNAcylation levels probably stem from the stress induced by the procedures (ischaemia–reperfusion, echocardiography), rather than being directly attributed to the anaesthesia itself.

## Methods

### Reagents

O-GlcNAcase inhibitor NButGT was synthesized using the Matthew S. Macauley method^39^.

### Animal model and measured parameters

The protocol was performed on 13-week-old male Wistar Han rats (Charles River Laboratory, les Oncins, France). The rats were delivered to the Experimental Therapy Unit and an acclimation period of one-week was observed. The rats were housed under standard conditions for temperature (21–24 °C), humidity (40–60%) and 12-h light/dark cycle with the light period starting at 7:00 a.m. Food and water were available ad libitum. Experiments were approved by and performed in accordance with the ethics committee in charge of animal experimentation of the Pays de la Loire (protocol #32744), French law on animal welfare, EU Directive 2010/63/EU for animal experiments, and the National Institutes of Health (NIH) Guide for the Care and Use of Laboratory Animals (NIH Pub. No. 85-23, revised 2011). Reporting is in accordance with current ARRIVE guidelines 2.0. Only males were used for this study to have easy access to a vein (penile vein) for injections. Abnormal breathing was considered as an exclusion criterion (breathing quality, absence of tremor). All of the rats received were included in the study. No confounding factors were identified in this study. Only the experimenter was aware of the group allocation at the different stages of the experiment.

**Figure 5:**
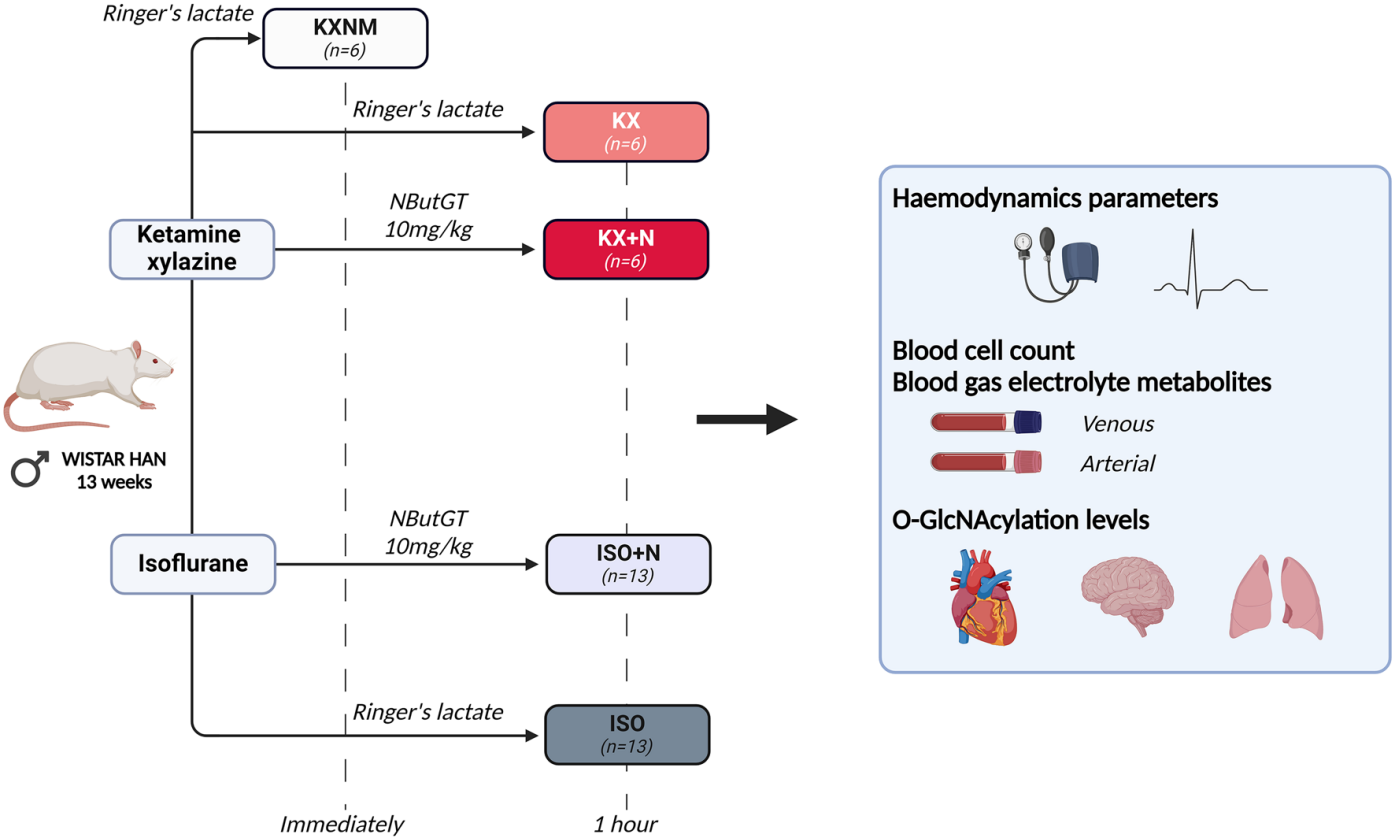
Timeline of the anaesthesia protocol and measured parameters. *KXNM* ketamine–xylazine anaesthesia non-maintained, *KX* ketamine–xylazine anaesthesia, *KX + N* ketamine–xylazine anaesthesia plus NButGT injection, *ISO* isoflurane anaesthesia, *ISO + N* isoflurane anaesthesia plus NButGT injection. Created with BioRender.com.

The rats were randomly anaesthetized either by intraperitoneal injection of ketamine (100 mg/kg)-xylazine (10 mg/kg) at a final volume of 1 mL/kg (n = 18), or by isoflurane (5% isoflurane, airflow rate 1L/min) (n = 26)^40,41^. The depth of anaesthesia was evaluated every 15 min via pedal reflex. Isoflurane anaesthesia was maintained with 2.5% isoflurane, air flow rate 0.6L/min. Ketamine-xylazine anaesthesia was maintained with half-dose injections (ketamine: 50 mg/kg, xylazine: 5 mg/kg, final volume 0.5 mL/kg) if the animal showed signs of lightness (paw withdrawal during pedal reflex, vibrissal movement, acceleration of respiratory rate). After induction, the rats were randomly injected intravenously through the penile vein with balanced solution (Ringer's lactate—1 mL/kg; Fresenius Kabi, Bad Homburg, Germany) (KX group; n = 12; ISO group; n = 13) or NButGT (10 mg/kg to 1 mL/kg) to increase O-GlcNAcylation levels (KX + N group; n = 6; ISO + N group; n = 13). In order to eliminate the impact of prolonged anaesthesia impregnation vis-à-vis the different assessed parameters, six rats in the KX group were sacrificed immediately after induction (KXNM group; n = 6) while the other groups were maintained on anaesthesia for 1 h (Fig. 5). Animal temperature was maintained within physiological values (37.5 to 38.5 °C) via a rectal probe coupled with a thermoregulated pad during the complete procedure. The animals were injected with lidocaine at the incision site for canulation of the carotid artery (subcutaneous injection before incision, lidocaine 2%, maximum 1.4 mL/kg) required for haemodynamic measurements.

Immediately (KXNM group) or 1 h (KX, KX + N, ISO, ISO + N) after induction of anaesthesia, haemodynamic parameters, blood gas (arterial), electrolyte and metabolite (venous) parameters with the epoc^®^ blood analysis system (Siemens Healthineers, Erlangen, Germany), as well as complete blood count (venous) with element HT5 (HESKA, Loveland, Colorado, USA) were evaluated. Haemodynamic parameters were assessed via canulation of the carotid artery and pressure transducer (IOX2 software, emka TECHNOLOGIES^®^, Paris, France).

The animals were euthanized by freeze clamping the beating heart to ensure preservation of post-translational modifications. After checking the depth of anaesthesia, lidocaine (subcutaneous injection before incision, lidocaine 2%, maximum 1.4 mL/kg) was injected at the incision sites. The thoracic cavity was then opened and the heart was clamped using Wollenberger forceps cooled in liquid nitrogen. In order to preserve post-translational modifications, other organs (lung and brain) were harvested and flash frozen in liquid nitrogen for biochemical analysis (Fig. 5).

### Tissue preparation

Frozen tissue was crushed in a mortar to obtain a powder to be used for protein analyses. In order to preserve post-translational modifications, all grinding steps were carried out in liquid nitrogen. The powder was then stored at −80 °C. Extraction for proteins from tissue was performed as previously described^15^.

### Western blot

Western blotting experiments were performed on nitrocellulose membrane as previously described^29,30^ (Supplementary Table S3). The amount of protein used for western blot was 25 µg. Analysis was performed using Image Lab software (Image Lab 6.1 version, Bio-Rad, CA, USA).

### Statistical analyses

Results are expressed as mean ± SEM of n different rats. Analyses of western blot are expressed in relation to the average of the protein quantification (stain free) and then reduced to the average of the control samples. The normal distribution of the data was tested with the D’Agostino & Pearson test. Data were analysed by one-way ANOVA with Fisher’s LSD test or Kruskal–Wallis test with uncorrected Dunn’s test according to whether the distribution was normal or not, respectively. A value of *p* < 0.05 was considered as significant. All statistical calculations and graphs were performed using GraphPad Prism software (version 8.4.2).

### Supplementary Information

Supplementary Information.

## Data Availability

The data that support the findings of this study are available from the corresponding author on reasonable request.
