# Supplementary material for: O-GlcNAcylation levels remain stable regardless of the anaesthesia in healthy rats
Source: Sci Rep. 2024 May 9;14:10669. doi: 10.1038/s41598-024-61445-0 (PMC11082205; doi:10.1038/s41598-024-61445-0)
Supplement: Supplementary file 1 — Supplementary Information. [file 41598_2024_61445_MOESM1_ESM.pdf]

O-GlcNAcylation levels remain stable regardless of the anaesthesia in healthy rats

Thomas Dupas[1,*], Amandine Vergnaud[1,*], Thomas Pelé[1], Angélique Blangy-Letheule[1], Virginie Aillerie[1], Martin Bouaud[1], Angélique Erraud[1], Anaïs Maillard[1], Dorian Hassoun[2], Antoine Persello[1], Jules Lecomte[2], Matthieu Rivière[3], Arnaud Tessier[3], Aurélia A. Leroux[1,4], Bertrand Rozec[2], Manon Denis[2], Benjamin Lauzier[1]

[1]Nantes Université, CNRS, INSERM, l'institut du thorax, F-44000 Nantes, France
[2]Nantes Université, CHU Nantes, CNRS, INSERM, l'institut du thorax, F-44000 Nantes, France
[3]Faculté des Sciences et des Techniques, Université de Nantes, CNRS, Chimie et Interdisciplinarité: Synthèse, Analyse, Modélisation (CEISAM), UMR CNRS 6230, Nantes, France
[4]Oniris, F-44300 Nantes, France

* The authors contributed equally to this work

Table S1. Impact of anaesthesia and NButGT treatment on O-GlcNAcylation-related enzymes

Cardiac O-GlcNAc-related enzymes	Group				
	KXNM	KX	KX+N	ISO	ISO+N
**OGT**	1.00 ± 0.13	1.14 ± 0.38	0.45 ± 0.05	0.57 ± 0.05	**0.70 ± 0.22$^{\#}$**
**OGA**	1.00 ± 0.11	0.79 ± 0.13	0.80 ± 0.21	1.15 ± 0.22	1.04 ± 0.17
**GFAT1**	1.00 ± 0.13	1.14 ± 0.38	0.45 ± 0.05	0.57 ± 0.05	0.70 ± 0.22
**GFAT2**	1.00 ± 0.11	0.77 ± 0.12	0.81 ± 0.49	0.73 ± 0.11	0.47 ± 0.13

Cerebral O-GlcNAc-related enzymes	Group				
	KXNM	KX	KX+N	ISO	ISO+N
**OGT**	1.00 ± 0.02	1.36 ± 0.33	1.35 ± 0.28	1.54 ± 0.32	1.15 ± 0.20
**OGA**	1.00 ± 0.08	1.20 ± 0.09	1.54 ± 0.22	1.37 ± 0.09	**1.11 ± 0.09$^{*, \#}$**
**GFAT1**	1.00 ± 0.14	1.37 ± 0.14	1.34 ± 0.28	1.36 ± 0.23	1.28 ± 0.19
**GFAT2**	1.00 ± 0.09	1.08 ± 0.06	0.83 ± 0.05	1.10 ± 0.14	1.13 ± 0.11

Pulmonary O-GlcNAc-related enzymes	Group				
	KXNM	KX	KX+N	ISO	ISO+N
**OGT**	1.00 ± 0.09	1.29 ± 0.24	1.74 ± 0.30	1.41 ± 0.17	1.26 ± 0.17
**OGA**	1.00 ± 0.09	1.15 ± 0.48	1.60 ± 0.72	1.32 ± 0.41	**2.03 ± 0.53$^{\#}$**
**GFAT1**	1.00 ± 0.06	1.06 ± 0.09	1.19 ± 0.35	0.84 ± 0.08	1.03 ± 0.17
**GFAT2**	1.00 ± 0.09	1.01 ± 0.12	0.88 ± 0.09	1.03 ± 0.06	1.01 ± 0.13

Cardiac, cerebral and pulmonary OGT, OGA, GFAT1 and GFAT2 protein expression were evaluated on rats under non-maintained anaesthesia (KXNM), ketamine-xylazine-maintained anaesthesia (KX), isoflurane anaesthesia (ISO), supplemented with NButGT (KX+N; ISO+N) to stimulate O-GlcNAcylation levels. Results expressed as mean ± SEM. Quantification of western blots are related to stain free. *: p<0.05 vs ISO; $^{\#}$: p<0.05 vs KX+N. KXNM (n=4-6); KX (n=4-6); KX+N (n=4-6); ISO (n=9-13); ISO+N (7-13). Cardiac OGT and GFAT1 are made on the same gel. Cardiac OGA and GFAT2 are made on the same gel.

Table S2. Impact of anaesthesia and NButGT treatment on venous cell blood count

	Group				
	KXNM	KX	KX+N	ISO	ISO+N
**White blood cells (10^9/L)**	6.79 ± 0.64	**3.22 ± 0.32***	4.39 ± 0.53	3.18 ± 0.35	**2.88 ± 0.27$^{\#}$**
**Lymphocytes (10^9/L)**	5.64 ± 0.54	**2.43 ± 0.27***	3.52 ± 0.48	2.50 ± 0.33	**2.25 ± 0.25$^{\#}$**
**Monocytes (10^9/L)**	0.24 ± 0.05	**0.15 ± 0.02***	0.20 ± 0.03	0.10 ± 0.01	**0.10 ± 0.01$^{\#}$**
**Neutrophils (10^9/L)**	0.88 ± 0.08	**0.62 ± 0.09***	0.65 ± 0.07	0.55 ± 0.05	0.50 ± 0.04
**Eosinophils (10^9/L)**	0.018 ± 0.004	0.016 ± 0.007	0.010 ± 0.004	**0.034 ± 0.007$^{\$}$**	**0.029 ± 0.005$^{\#}$**
**Basophils (10^9/L)**	0.010 ± 0.003	0.004 ± 0.002	0.002 ± 0.002	0.003 ± 0.001	0.003 ± 0.001
**Red blood cells (10^{12}/L)**	6.49 ± 0.07	6.30 ± 0.08	**6.69 ± 0.13$^{\$}$**	6.44 ± 0.08	6.48 ± 0.11
**Hemoglobinaemia (g/L)**	132 ± 1	132 ± 2	138 ± 2	130 ± 1	130 ± 2
**Platelets (10^9/L)**	628 ± 28	532 ± 70	731 ± 51$^{\$}$	682 ± 24$^{\$}$	585 ± 54

Complete blood count was evaluated on rats under non-maintained anaesthesia (KXNM), ketamine-xylazine-maintained anaesthesia (KX), isoflurane anaesthesia (ISO), supplemented with NButGT (KX+N; ISO+N) to stimulate O-GlcNAcylation levels. Results expressed as mean ± SEM. *: p<0.05 vs KXNM; $^{\#}$: p<0.05 vs KX + N; $^{\$}$: p<0.05 vs KX. KXNM (n=5); KX (n=5); KX+N (n=6); ISO (n=13); ISO+N (n=13).

Table S3. Antibodies used in Western blot analysis

Target	Primary antibody		Secondary antibody	
	Antibody	Dilution	Antibody	Dilution
**O-GlcNAc**	HRP Anti-O-linked N-Acetylglucosamine [RL2] Abcam #ab201995	1/10 000[#]	/	/
**GFAT1**	GFAT1 (D12F4) Cell Signaling #5322	1/500*	Anti-rabbit IgG, HRP-linked antibody Cell Signaling #7074	1/10000*
**GFAT2**	Anti-GFPT2 [EPR19095] Abcam #ab190966	1/2000*	Anti-rabbit IgG, HRP-linked antibody Cell Signaling #7074	1/10000*
**OGT**	OGT (D1D8Q) Cell Signaling #24083	1/400*	Anti-rabbit IgG, HRP-linked antibody Cell Signaling #7074	1/10000*
**OGA**	Anti-MGEA/OGA Abcam #ab105217	1/5000*	Anti-rabbit IgG, HRP-linked antibody Cell Signaling #7074	1/10000*

Dilutions carried out in 3% Bovine Serum Albumin ([#]) or in 5% milk (*).

Cardiac O-GlcNAc

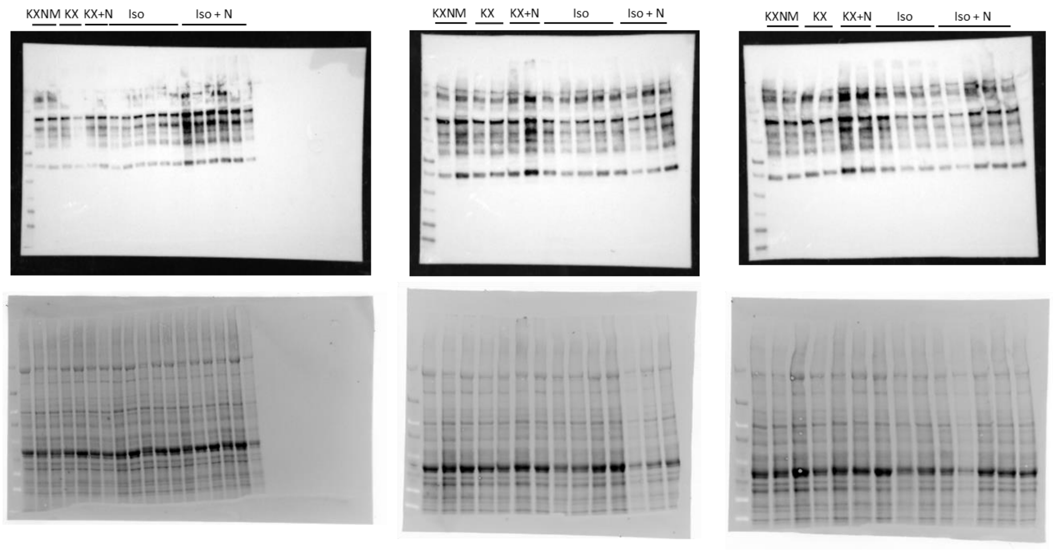

Cerebral O-GlcNAc

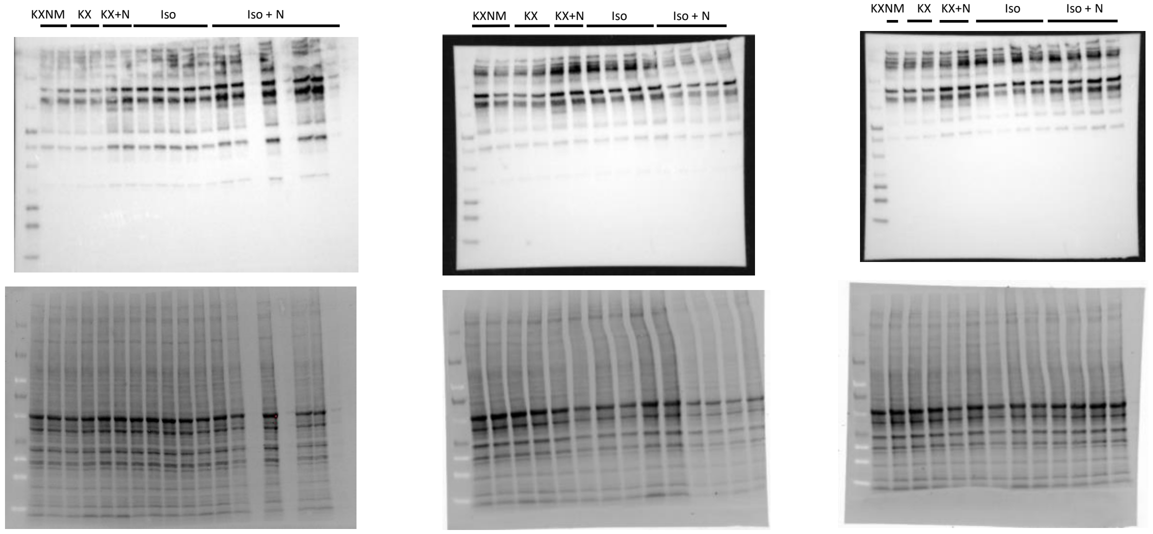

Pulmonary O-GlcNAc

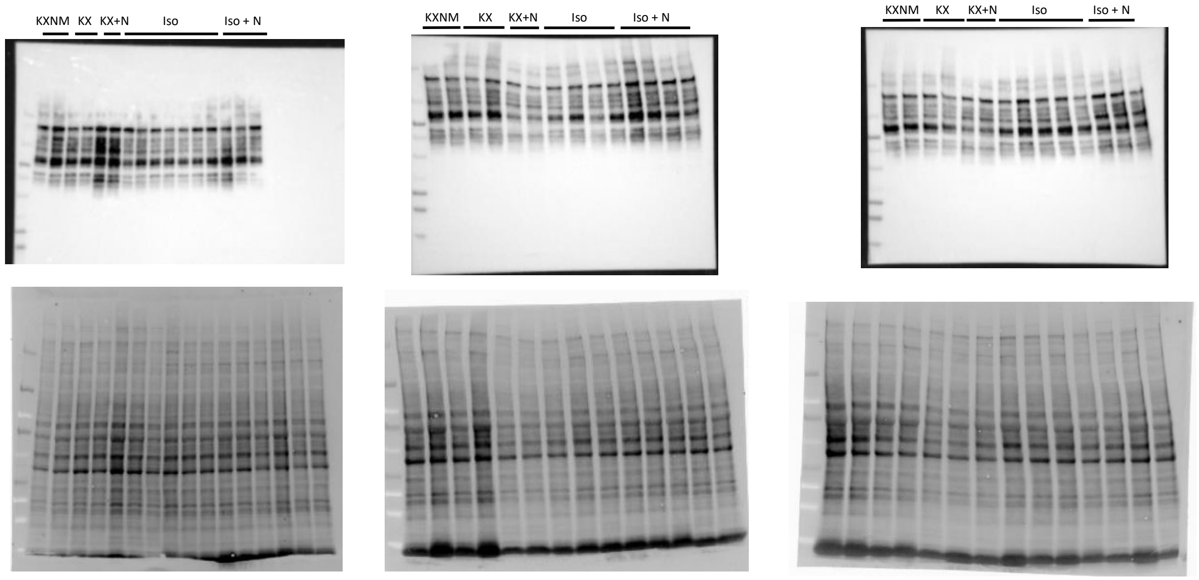

Figure S1

Cardiac OGT

Cardiac GFAT1

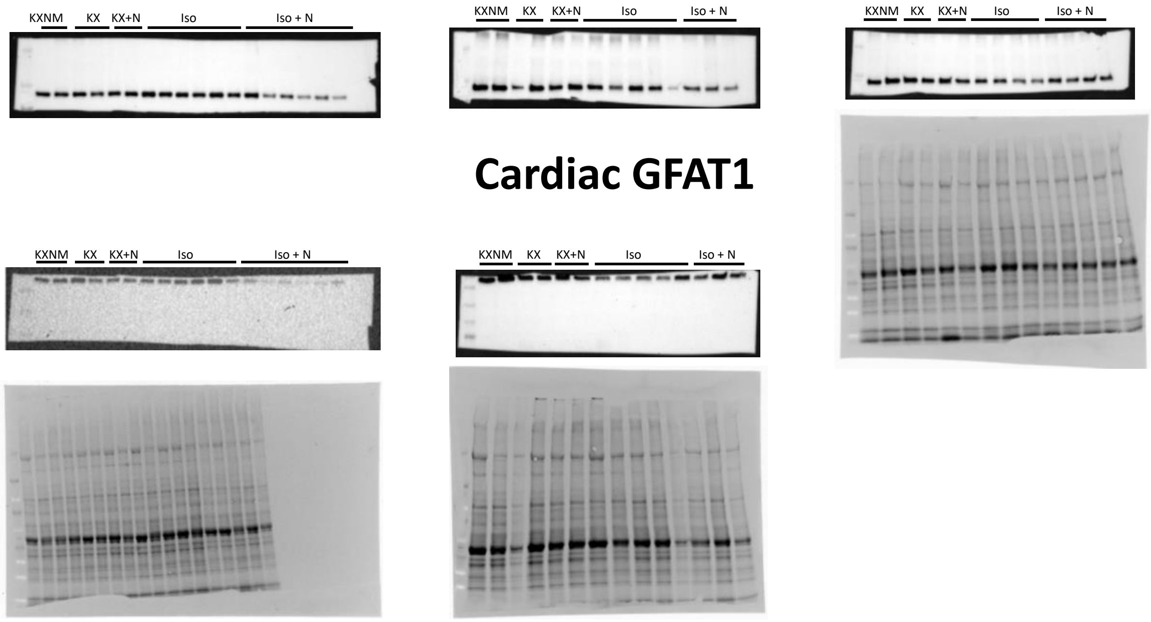

Cardiac OGA

Cardiac GFAT2

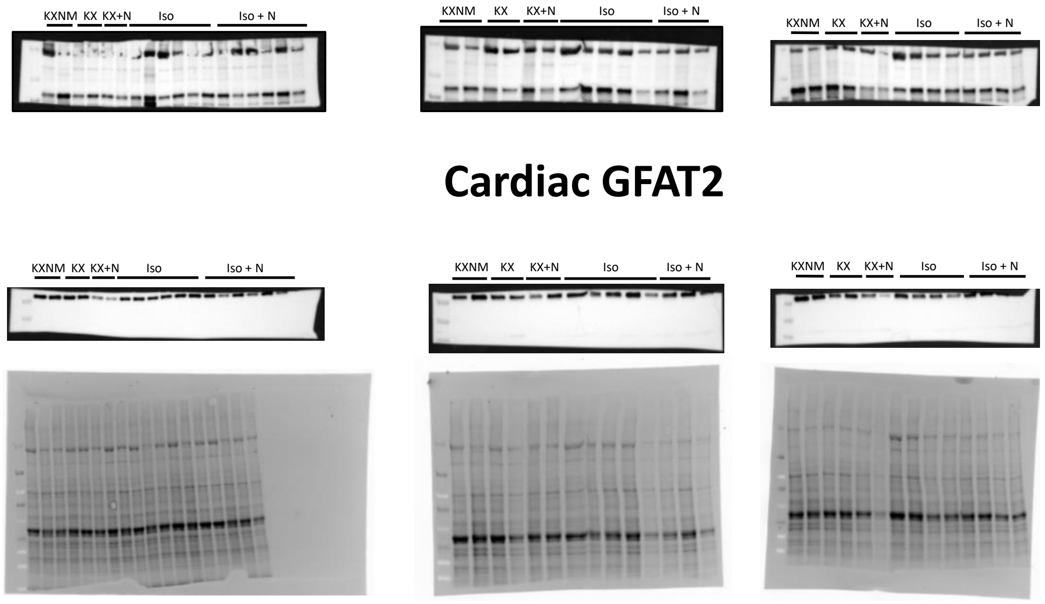

Figure S2

Cerebral OGT

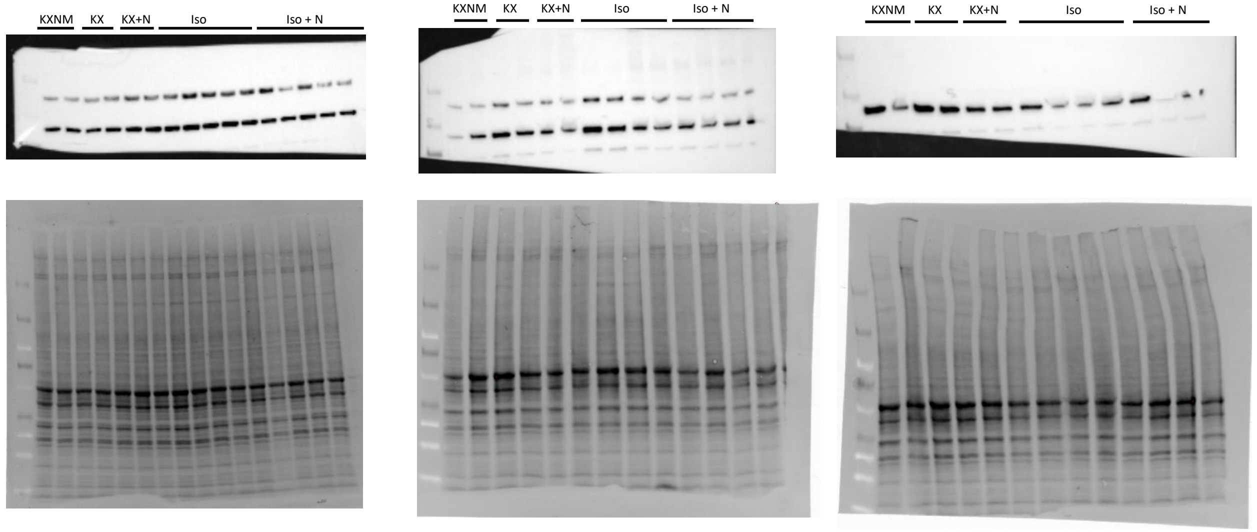

Cerebral OGA

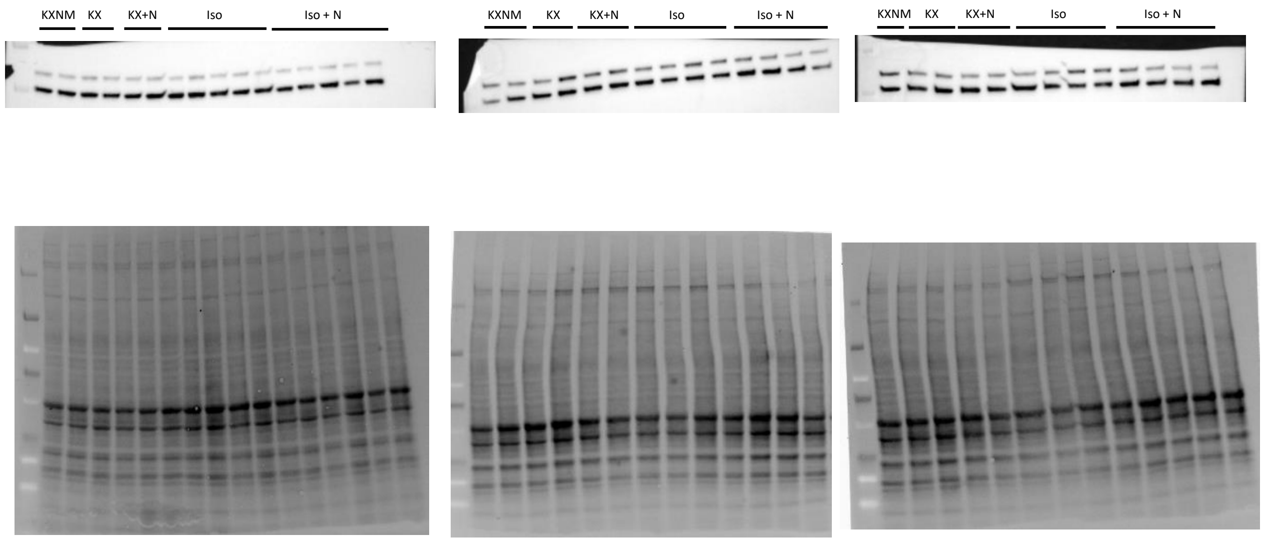

Cerebral GFAT1

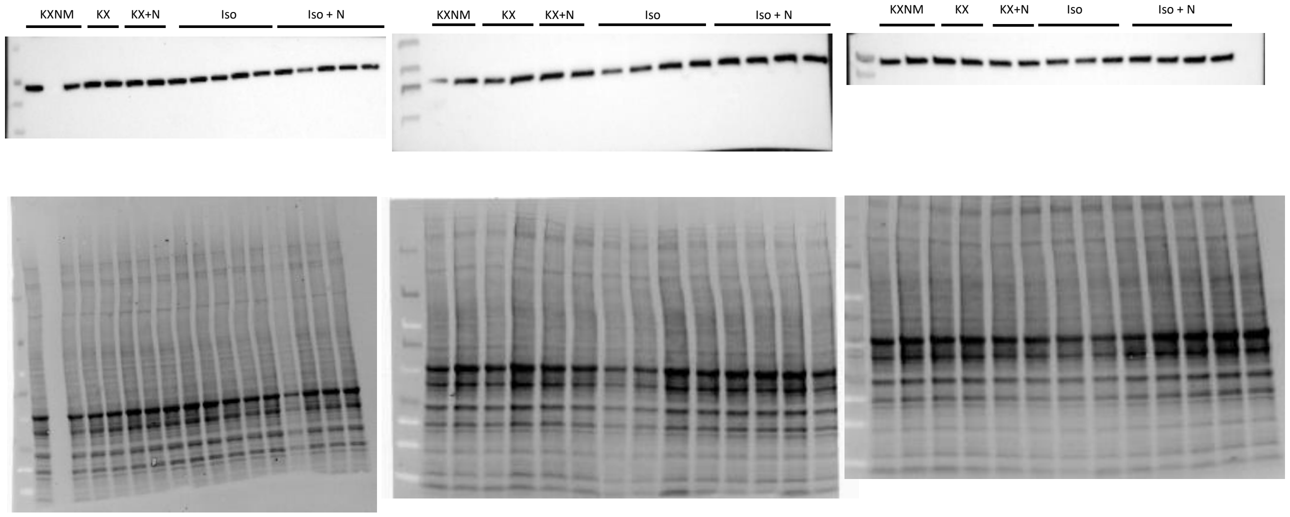

Cerebral GFAT2

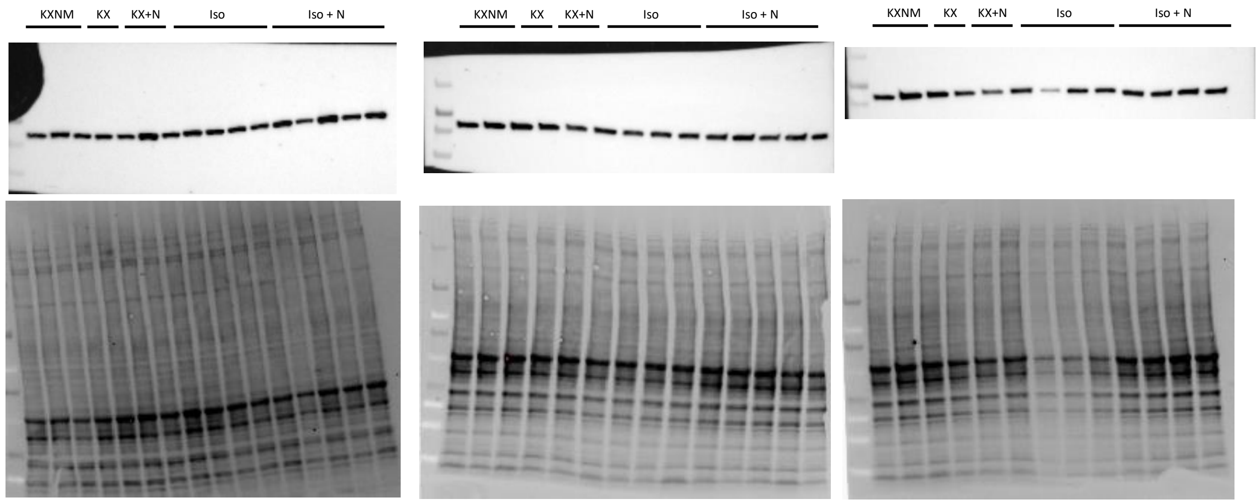

Figure S3

Pulmonary OGT

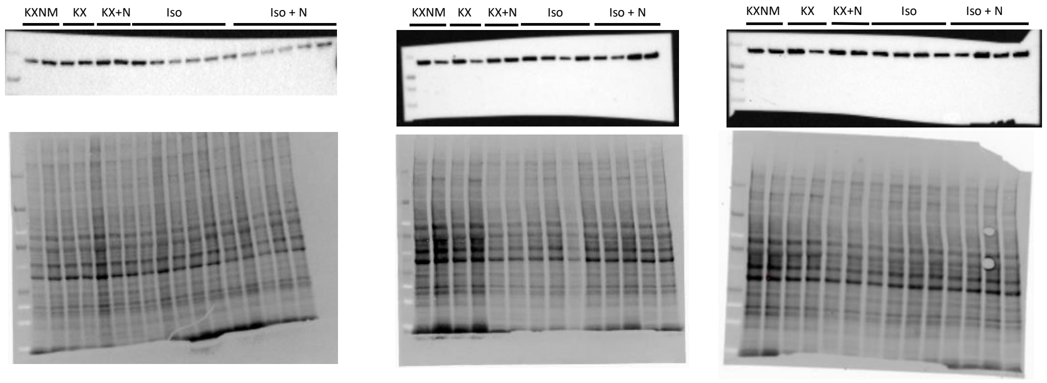

Pulmonary OGA

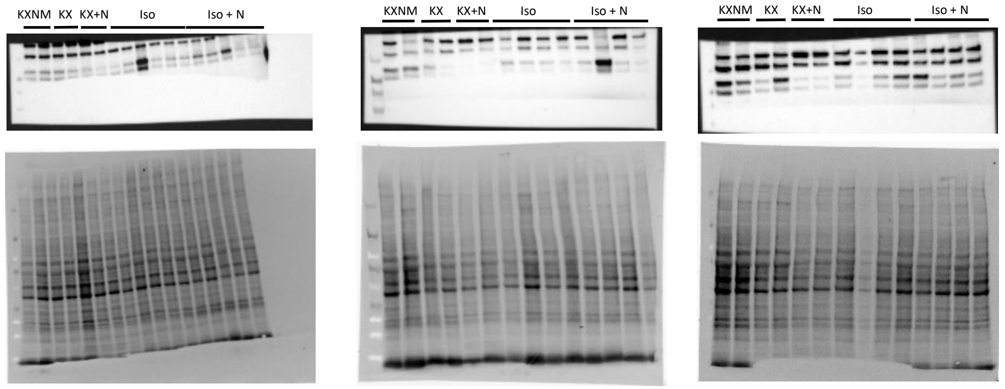

Pulmonary GFAT1

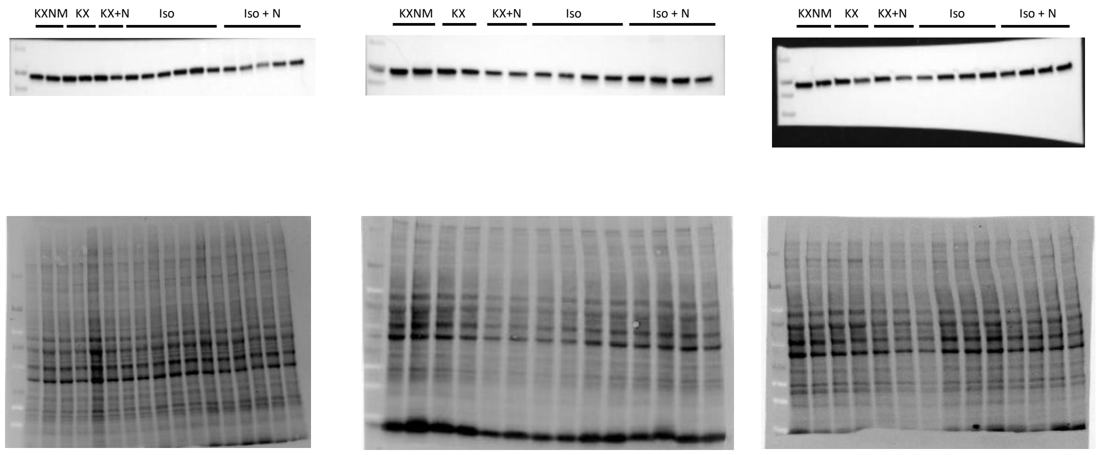

Pulmonary GFAT2

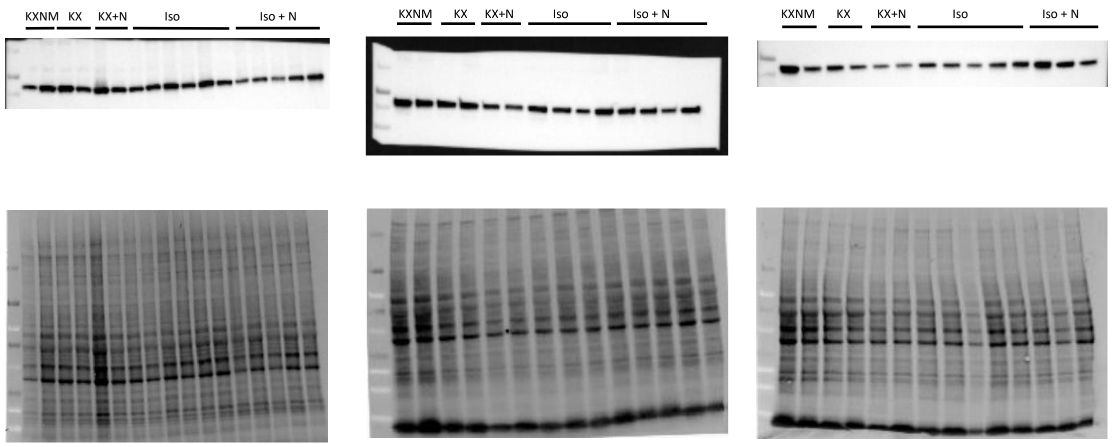

Figure S4
